# Serum Levels of S100A8/A9 as a Biomarker of Disease Activity in Patients with IgA Vasculitis

**DOI:** 10.3390/biomedicines12040750

**Published:** 2024-03-28

**Authors:** Sasa Srsen, Martina Held, Mario Sestan, Nastasia Kifer, Ana Kozmar, Daniela Supe Domic, Benjamin Benzon, Alenka Gagro, Marijan Frkovic, Marija Jelusic

**Affiliations:** 1 Department of Paediatrics, University Hospital Split, Spinciceva 1, 21000 Split, Croatia; ssrsen@kbsplit.hr; 2 School of Medicine, University of Split, Soltanska 2, 21000 Split, Croatia; benzon.benjamin@gmail.com; 3 Department of Paediatrics, University Hospital Centre Zagreb, Kispaticeva 12, 10000 Zagreb, Croatiamfrkovic1@gmail.com (M.F.); 4 School of Medicine, University of Zagreb, Salata 3, 10000 Zagreb, Croatia; ana.kozmar@kbc-zagreb.hr (A.K.); alenka.gagro@gmail.com (A.G.); 5 Department of Laboratory Diagnostics, University Hospital Centre Zagreb, Kispaticeva 12, 10000 Zagreb, Croatia; 6 Department of Medical Laboratory Diagnostic, University Hospital Split, Spinciceva 1, 21000 Split, Croatia; dsupe@kbsplit.hr; 7 Department of Health Studies, University of Split, Ulica Rudjera Boskovica 35, 21000 Split, Croatia; 8 Department of Paediatrics, Children’s Hospital Zagreb, Ulica Vjekoslava Klaica 16, 10000 Zagreb, Croatia; 9 Medical Faculty Osijek, Josip Juraj Strossmayer University of Osijek, Josipa Huttlera 4, 31000 Osijek, Croatia

**Keywords:** leukocyte L1 antigen complex, IgA vasculitis, biomarkers, child, adolescent

## Abstract

S100A8/A9 protein is a well-known marker of disease activity or severity in many autoimmune and autoinflammatory diseases, but there have not been many studies about the role of S100A8/A9 in IgA vasculitis (IgAV). The aim of our study was to evaluate S100A8/A9 as a possible biomarker of activity in IgAV. We measured the serum levels of S100A8/A9 in pediatric patients with IgA vasculitis at the onset of the disease, after three months, and after six months. We compared these levels between patients with active disease, remission, and a control group, and assessed their correlation with disease activity and other markers of inflammation. Patients with active disease had significantly higher levels of serum S100A8/A9 (median ± SD) than those in the control group at the beginning of the disease (5740 ± 3157 ng/mL vs. 1447 ± 858.3 ng/mL; *p* < 0.0001), but also three months and six months after disease onset (*p* < 0.001). There was a positive correlation between S100A8/A9 serum levels and disease activity (*p* = 0.0003). Patients with active disease had significantly higher levels of S100A8/A9 than those in remission three months after disease onset (*p* = 0.0260). There was a correlation between S100A8/A9 and C-reactive protein, the C3 component of complement, ferritin, and fibrinogen. Serum levels of S100A8/A9 were also higher in patients with greater skin areas covered with rash. We demonstrated that serum levels of S100A8/A9 correlated well with disease activity and other biomarkers of inflammation in children with IgAV. According to our results, serum S100A8/A9 may be a good indicator of active disease in IgAV.

## 1. Introduction

Immunoglobulin A vasculitis (IgAV), formerly known as Henoch–Schönlein purpura, is the most common vasculitis in the pediatric population [1]. It presents with a wide range of symptoms such as rash, joint pain, kidney and gastrointestinal tract disease, and, less often, other organ involvement [2]. According to the European Alliance of Associations for Rheumatology (formerly known as the European League against Rheumatism) (EULAR)/Paediatric Rheumatology International Trials Organisation (PRINTO)/Paediatric Rheumatology European Society (PRES) classification criteria, a patient with IgAV needs to have non-thrombocytopenic palpable purpura and petechiae, with at least one of the following four criteria: abdominal pain, arthritis or arthralgia, renal involvement and leukocytoclastic vasculitis with predominant IgA deposits, or proliferative glomerulonephritis with predominant IgA deposits [3].

S100A8/A9 is a heterodimer of S100A8 and S100A9 proteins, which are members of the S100 protein family. It is also known as calprotectin, MRP8/MRP14 (myeloid-related-protein 8/14), calgranulin A/B, L1 protein and cystic fibrosis antigen [4]. It is expressed in neutrophils, monocytes, and early differentiated macrophages [5,6] and has multiple intracellular and extracellular functions. Intracellularly, the calcium-dependent interaction of S100A8/A9 complex with cytoskeletal components such as microtubules, vimentin, keratin, and actin filament has an important role in phagocyte transendothelial migration. While extracellularly, it expresses antimicrobial activity, probably linked to its zinc molecules, promotes endothelial activation, impairment of endothelial monolayer integrity, apoptosis and necrosis of endothelial cells, as well as chemotaxis of neutrophils, inducing proinflammatory effects [4,7]. Inside cells, it regulates cell proliferation, differentiation, calcium homeostasis, the arachidonic acid metabolism, cytoskeletal modulation, and subsequent cell migration. However, once released into the extracellular space, S100A8/A9 acts as alarmin after binding to different receptors, most importantly receptors for advanced glycation end products (RAGE) and Toll-like receptors 4 (TLR4), and this interaction triggers nuclear factor kappa B (NF-κB) signaling pathways and, thus, the synthesis of proinflammatory cytokines [4,5,6,7]. The release of tumor necrosis factor alpha (TNF-α), interleukin–1β (IL-1β), and interleukin-6 (IL-6) promotes chemotaxis and leukocyte recruitment. Alongside this, S100A8/A9 also stimulates cytokine secretion acting in a directly proinflammatory way. But on the other hand, it may also exert an anti-inflammatory role in specific conditions when there is a threat of tissue damage caused by intensive inflammation. By acting in this way, S100A8/A9 has a dual role in maintaining homeostasis [4,5,6,7]. S100A8/A9 also has a role in the resistance to pathogens by attenuating bacterial adhesion and invasion. Inflammatory processes such as infection, trauma, heat, stress, and others intensively increase levels of S100A8/A9 in those cells. Different types of inflammation may induce upregulation of S100A8/A9 production, not only immune system related conditions including autoimmune diseases and hypersensitivity reactions, but also infections, metabolic inflammation such as gout, diabetes, or obesity, or inflammatory processes in degenerative diseases, for example, osteoarthritis [4,5,6,7].

It has been shown that S100A8/A9 can be a useful biomarker of activity of disease or predictor of severity of disease in various rheumatic diseases, such as juvenile idiopathic arthritis (JIA), rheumatoid arthritis (RA), systemic lupus erythematosus (SLE), anti-neutrophil cytoplasmic antibodies-associated vasculitis (AAV), lung fibrosis in systemic sclerosis, adult-onset Still’s disease, Kawasaki disease (KD), or autoinflammatory diseases [4,7,8,9,10,11,12]. However, there are not many data about the possible role of S100A8/A9 in IgAV.

The aim of our study was to evaluate the possible role of the serum level of S100A8/A9 as a biomarker of disease activity in patients with IgAV.

## 2. Results

Data were collected from 69 patients with IgAV (36 boys and 33 girls), with mean (range) age of 6.4 (3.7–9.1) years (Table 1). Boys were slightly more prevalent than girls in a ratio of 1.1:1. We compared patients with active disease and in remission, as well as with a control group of 33 patients (21 boys and 12 girls), with mean (range) age of 6.6 (3.8–9.2).

The onset of IgA vasculitis in patients was predominantly marked by the emergence of a purpuric rash, observed in 98.6% of cases (68 patients), with only one patient developing the rash later in the disease course. A total of 56 patients had joint pain or arthritis at some point of the disease (81.2%). A total of 26 of them (37.7%) had gastrointestinal involvement (abdominal pain, positive fecal occult bleeding test), while 18 patients (26.1%) had nephritis presenting with hematuria, proteinuria, or a combination of both (Table 1).

In most of the patients, remission occurred early during the disease, so only nine patients (13.0%) had active disease three months after onset of disease, and five of them (7.2%) still did not achieve remission six months after the beginning of the disease (Table 1).

A total of 61 patients (88.4%) received at least some kind of treatment. Most often, they were treated with nonsteroidal anti-inflammatory drugs (42 patients; 60.9%) and glucocorticoids (35 patients; 50.7%), while a small number of patients were treated with immunosuppressant and immunomodulatory drugs, such as mycophenolate mofetil, cyclophosphamide, or intravenous immunoglobulins (six patients; 8.7%). Nine patients (13.0%) were treated with antibiotics due to concomitant infections. Also, 48 patients were treated with other drugs (ACE inhibitors, proton pump inhibitors H2 histamine receptor antagonists, symptomatic therapy; Table 1).

**Figure 1:**
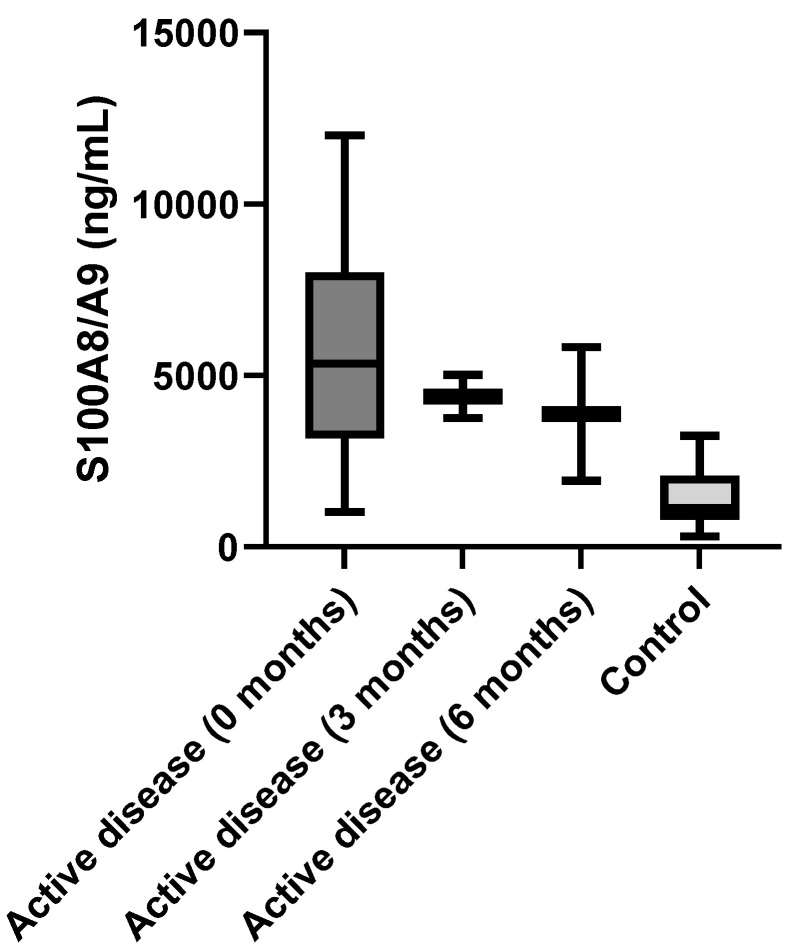
Serum S100A8/A9 levels in patients with active IgA vasculitis at the beginning of the disease, three months after beginning, six months after beginning, and the control group.

There was a significant difference in S100A8/A9 serum level between patients with active IgAV at the beginning of the disease and the control group (5740 ± 3157 ng/mL vs. 1447 ± 858.3 ng/mL; *p* < 0.0001). We observed significant differences between patients with active IgAV and the control group at other time points—three months and six months after the onset of the disease (0 months: 5740 ± 3157 ng/mL; three months: 4386 ± 899.4 ng/mL; six months: 3887 ± 2758 ng/mL; control group: 1447 ± 858.3 ng/mL; R^2^ = 0.365; *p* < 0.001) (Figure 1).

**Figure 2:**
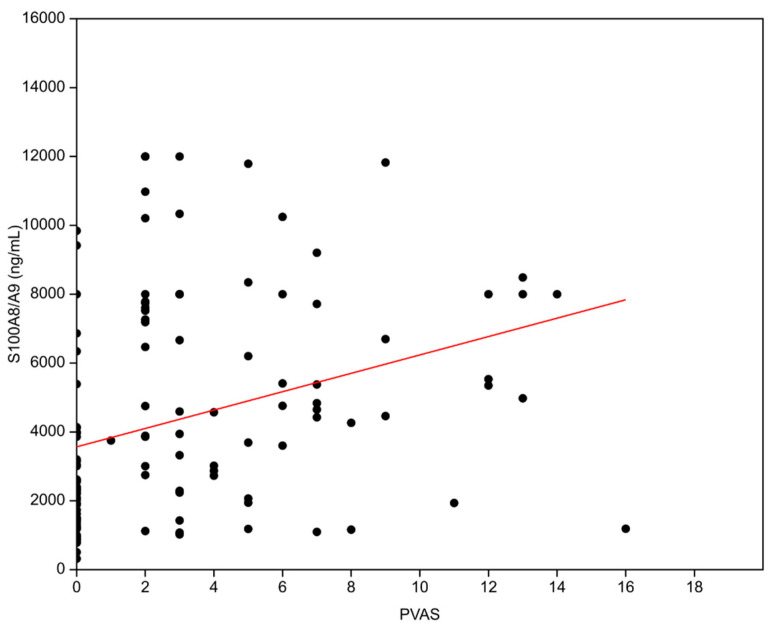
Correlation between Pediatric Vasculitis Activity Score (PVAS) and serum S100A8/A9 levels (R^2^ = 0.1006, *p* = 0.0003).

There was a correlation present between S100A8/A9 serum levels and PVAS score (ß_0_ = 3566.9, ß_1_ = 266.98, R^2^ = 0.1006, *p* = 0.0003, Figure 2). Patients with a more active disease tend to have more elevated S100A8/A9 serum levels. The connection with disease activity is additionally reflected in the fact that in patients in whom the disease was persistently active, the values of S100A8/A9 remained elevated over time.

**Figure 3:**
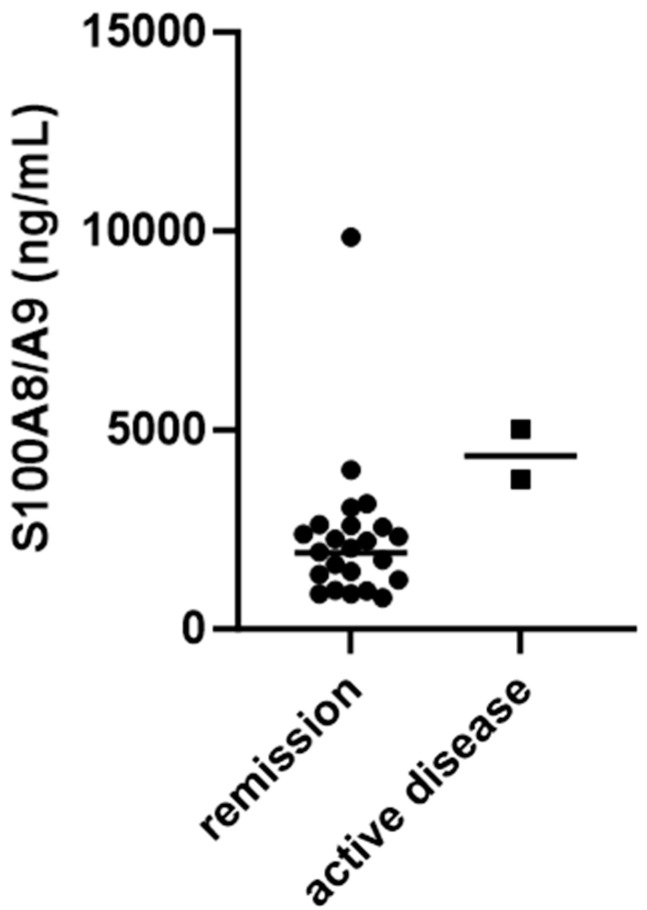
Serum S100A8/A9 levels in patients in remission and those with active IgA vasculitis three months after beginning of disease.

Namely, when looking at S100A8/A9 serum levels in patients with active disease and those in remission, a statistically significant difference was found in patients tested three months after the beginning of the disease (4386 ± 899.4 ng/mL vs. 2294 ± 1846 ng/mL, *p* = 0.0260) (Figure 3).

**Figure 4:**
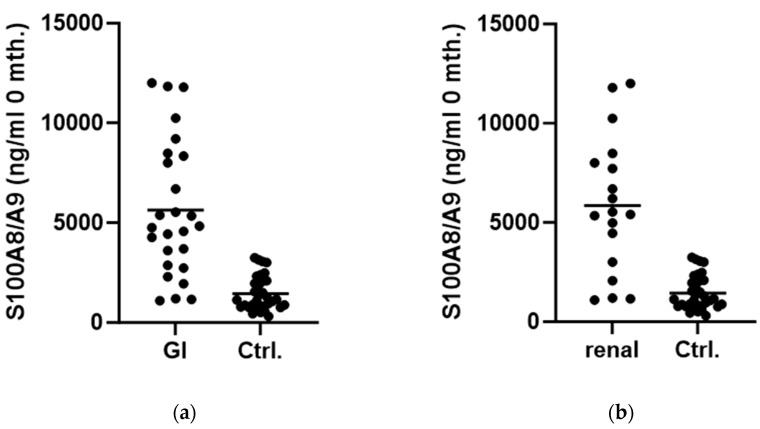
Comparison between serum S100A8/A9 levels in patients at onset of disease and control group: (**a**) in patients with gastrointestinal involvement; (**b**) in patients with renal disease. (Abbreviations: GI—gastrointestinal involvement. Ctrl.—Control group.).

There was a difference in values of serum S100A8/A9 present when we separately compared the group of patients with gastrointestinal involvement with the control group (5626 ± 3395 ng/mL vs. 1447 ± 858.3 ng/mL; *p* < 0.0001, Figure 4a). This was the same as when we compared patients with renal disease with the control group (5855 ± 3433 ng/mL vs. 1447 ± 858.3 ng/mL; *p* < 0.0001, Figure 4b).

Comparing levels of S100A8/A9 in groups of patients with specific clinical manifestations, we found no difference between patients with nephritis and those without renal disease (*p* = 0.5858), patients with gastrointestinal involvement and those without it (*p* = 0.8147), or patients with or without joint affection (*p* = 0.7269).

**Figure 5:**
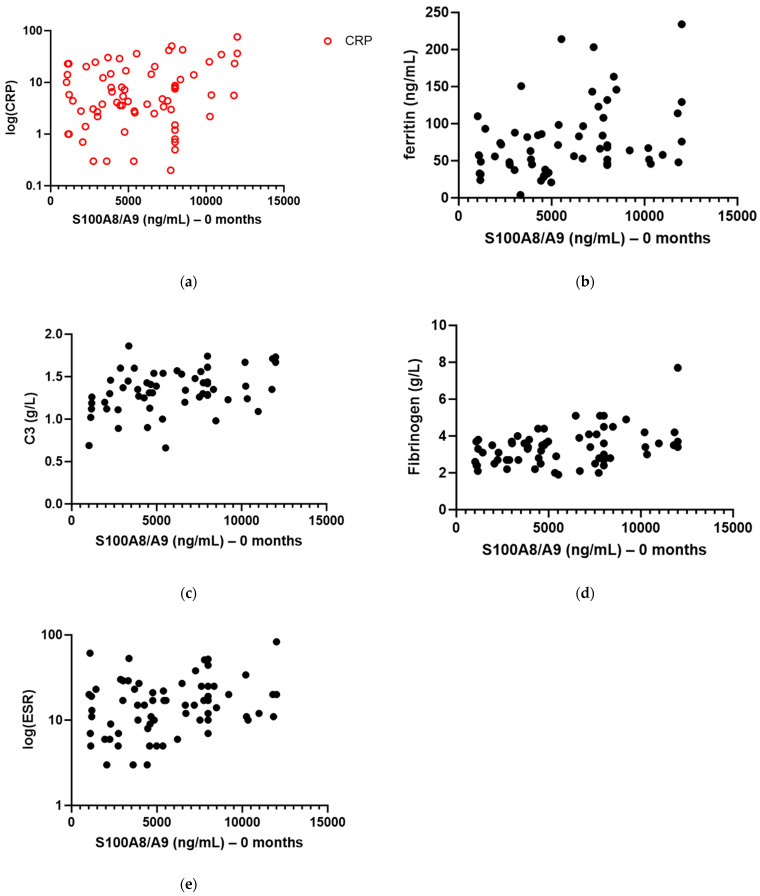
Correlation between serum S100A8/A9 level in patients with active IgA vasculitis at onset of disease and other biomarkers of inflammation: (**a**) C-reactive protein (log-transformed) (R^2^ = 0.1030, *p* = 0.0076); (**b**) ferritin (R^2^ = 0.1143, *p* = 0.0077); (**c**) C3 component of complement (R^2^ = 0.1294, *p* = 0.0065); (**d**) fibrinogen (R^2^ = 0.1408, *p* = 0.0019); (**e**) erythrocyte sedimentation rate (log-transformed) (R^2^ = 0.0488, *p* = 0.0725).

We found a correlation between S100A8/A9 serum level and CRP (ß_0_ = 3.193, ß_1_ = 0.001509, R^2^ = 0.1030, *p* = 0.0076), ferritin (ß_0_ = 47.72, ß_1_ = 0.004954, R^2^ = 0.1143, *p* = 0.0077), C3 (ß_0_ = 1.163, ß_1_ = 0.000029, R^2^ = 0.1294, *p* = 0.0065), and fibrinogen serum levels (ß_0_ = 2.723, ß_1_ = 0.000113, R^2^ = 0.1408, *p* = 0.0019) in patients with active disease (Figure 5a–d). The correlation with ESR (Figure 5e) was close to statistical significance (ß_0_ = 12.76, ß_1_ = 0.001071, R^2^ = 0.0488, *p* = 0.0725).

No correlation was found between serum S100A8/A9 level and white blood cell count (WBC) (*p* = 0.6514), C4 component of complement (*p* = 0.3843), albumin (*p* = 0.9029), or gamma-globulin level (*p* = 0.8788). The descriptive statistics of the tested markers of inflammation are shown in Table 2.

When we were comparing levels of inflammatory markers at the beginning of the disease, after three months and after six months in patients with active disease, we found no difference between S100A8/A9 and CRP levels dynamics (*p* = 0.4031), as well as between S100A8/A9 and ESR (*p* = 0.2381).

We found no correlation between serum and fecal S100A8/A9 levels, either among all patients with IgAV (*p* = 0.2747) or in the group of patients with gastrointestinal involvement, separately (*p* = 0.5139). There were significantly higher levels of fecal S100A8/A9 in patients with gastrointestinal involvement (50 ± 169.9 µg/g vs. 27.5 ± 49.1 µg/g, *p* = 0.0099).

When we were looking for other markers of inflammation in patients with active IgAV, we also found that they had significantly higher levels of CRP than those in the control group (11.71 ± 1.759 mg/L vs. 0.55 ± 0.3008 mg/L, *p* < 0.0001), as well as WBC (11.0 ± 3.93 × 10^9^/L vs. 5.872 ± 1.707 × 10^9^/L, *p* < 0.0001), ESR (18.78 ± 15.73 mm/h vs. 6.7 ± 4.095 mm/h, *p* < 0.0001), and ferritin (76.44 ± 47.7 μg/L vs. 44.89 ± 43.56 μg/L, *p* = 0.0018).

**Figure 6:**
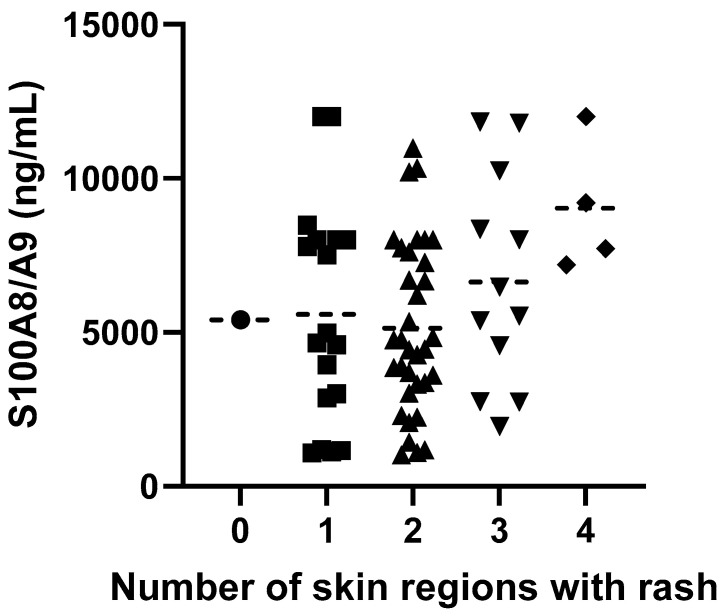
Serum S100A8/A9 levels depending on number of skin regions covered with rash.

Patients with active disease who had three or more skin regions (legs, trunk, arms, head, and neck) covered with a rash had higher S100A8/A9 serum levels than those with a rash in two or less regions (no rash: 5409 ± 0 ng/mL; rash in one region: 5577 ± 3556 ng/mL; rash in two regions: 5136 ± 2765 ng/mL; rash in three regions: 6631 ± 3448 ng/mL; rash in four regions: 9027 ± 2158 ng/mL; R^2^ = 0.09, *p* = 0.0376) (Figure 6).

Levels of serum S100A8/A9 after six months in patients who had a flare up of the disease were higher than in those without a flare up, but not statistically significant, although close to the threshold (5257.7 ± 1774.3 ng/mL vs. 2765.7 ± 416.3 ng/mL; *p* = 0.0919).

## 3. Discussion

S100A8/A9 has an important role in innate immunity. It is mainly produced by cells that participate in inflammation, such as neutrophils and monocytes. Inflammatory processes such as infection, trauma, heat, stress, and others, intensively increase levels of S100A8/A9 in those cells. Different types of inflammation may induce upregulation of S100A8/A9 production, not only immune system-related conditions including autoimmune diseases and hypersensitivity reactions, but also infections, metabolic inflammation such as gout, diabetes, or obesity, or inflammatory processes in degenerative diseases, for example, osteoarthritis [13].

The role of S100A8/A9 in IgAV has not been thoroughly investigated yet. Kawasaki et al. showed that patients with IgAV who had higher serum S100A8/A9 levels also had more severe renal disease and suggested that serum S100A8/A9 levels may be associated with the severity of renal disease in patients with IgAV nephritis (IgAVN), but also noticed that further studies are required [14].

The most important result of our study was that serum S100A8/A9 correlated with disease activity in patients with IgAV and that in patients who have not achieved remission of the disease, the levels remain elevated compared to patients with inactive disease, even three months after the onset of IgAV. Since the treatment is aimed at those patients who have active disease, measuring serum S100A8/A9 levels could have a function in determining the length of treatment or when the treatment can be stopped. Given that a particle-enhanced turbidimetric immunoassay can be employed to detect the S100A8/A9 heterodimer in biological samples, and this method is adaptable for use with automated standard analyzers, it ensures a dependable clinical measurement of S100A8/A9. Consequently, incorporating this procedure into routine clinical practice can be seamlessly achieved. However, taking into account the relatively small number of patients in whom the activity of the disease persisted, that is, who did not achieve remission even three months after the onset of the disease in our study, in order to draw definitive conclusions, it is necessary to conduct research on a larger number of patients.

Although in our study patients with renal disease had higher levels of S100A8/A9 in serum than the control group, and similar results were found in the group of IgAV patients with gastrointestinal involvement, we found no differences between patients with active disease when we were comparing those with renal disease with ones without it, similar to the comparison of those with or without gastrointestinal or joint involvement. The results of our research, therefore, did not show that serum S100A8/A9 is a predictor of nephritis.

Previously, it was demonstrated that during the active phase of antineutrophil cytoplasmic antibody (ANCA) associated vasculitis (AAV), both monocytes and neutrophils are activated [7]. These patients exhibit elevated levels of cell surface S100A8/A9 on both neutrophils and monocytes in comparison to healthy controls, and these levels further increase during active disease. Additionally, serum S100A8/A9 levels mirror these cellular findings, showing significantly higher levels in active disease compared to remission and those found in healthy controls. Conversely, in patients with various glomerulonephritides, glomerular macrophage expression of S100A8/A9 was observed. Concerning the role of macrophages in IgAVN, the accumulation of macrophages in kidneys affected by glomerulonephritis has been reported to be associated with the inflammatory activity of the glomerulonephritis [15]. The possible reason why in our study we did not find a difference between patients with IgAVN compared to other patients with IgAV is, therefore, that in patients with IgAV, regardless of the clinical phenotype of the disease, and similar to patients with AAV, neutrophils in the blood could be activated (which is associated with an increase in the serum level of S100A8/A9), and in patients with IgAVN there is an additional accumulation of macrophages in the kidney and an increase in the expression of S100A8/A9 on glomerular macrophages. Therefore, the measurement of serum S100A8/A9 levels cannot distinguish IgAV patients who have nephritis from those who have other clinical manifestations of the disease. Perhaps the measurement of S100A8/A9 activity in the kidney itself, i.e., in the kidney biopsy, could contribute to determining the severity of nephritis in IgAV and/or its prognosis.

Patients in our study had significantly higher serum S100A8/A9 levels when three or more skin regions were covered with a rash. A similar correlation between serum S100A8/A9 levels and skin rash presence was found in patients with systemic-onset JIA [16]. It has been shown that skin stress upregulates S100A8 and S100A9 genes with elevated levels of proteins shown in ex vivo skin culture after skin stress [17]. Since the skin is an organ of remarkable size, it is likely to be responsible for the noticed rise in serum levels of S100A8/A9 [4]. In our prior studies, we demonstrated an association between an enlarged rash in patients with IgAV, more severe GI manifestations [18], and an increased risk of developing nephritis [19].

S100A8/A9 serum levels were shown to have significance in patients with other types of vasculitis. Patients with Kawasaki disease, with the active disease, had higher levels of serum S100A8/A9 than those in remission and although it had no predictive values of intravenous immunoglobulin response, it was shown to be a good marker of disease activity, and persistently elevated levels in serum were associated with a higher risk of developing coronary artery aneurisms [20,21]. There was also an association between serum S100A8/A9 levels and disease activity or risk of relapses in patients with AAV [7,11,12]. In our study, the association between the level of serum S100A8/A9 and relapse in patients with IgAV was at the limit of statistical significance.

The role of serum S100A8/A9 has been more comprehensively investigated in other rheumatic diseases.

In patients with JIA, a correlation between serum S100A8/A9 level and disease activity was shown. Patients with active disease had higher levels of S100A8/A9 than those in remission and also with clinical markers of the disease [22,23]. In our study, we showed that patients with active IgAV also have higher levels of serum S100A8/A9 than those in remission as well as those in the control group. Berntzen et al. described a correlation between S100A8/A9 levels and other laboratory markers of inflammation such as CRP and ESR, with S100A8/A9 having an even stronger correlation with disease activity [23]. Therefore, it can be considered as a sensitive marker of local inflammation even in patients without clinical signs of disease activity and it can be used to predict potential flare ups of the disease [24,25]. One of the patients in our study, who was in clinical remission after three months of having the disease, had significantly higher levels of S100A8/A9 than all other patients both in remission and with active disease. Since that patient did not have any other clinical symptoms of active disease, or laboratory results that would suggest he had an active inflammatory process, it is possible that this patient had not achieved laboratory remission, i.e., that smoldering inflammation is still present. This is exactly the situation described in the literature in the example of JIA [24,25].

Our group of patients with IgAV presented a correlation between S100A8/A9 levels in serum and CRP as well as some other markers of inflammation such as ferritin, C3, and fibrinogen. The correlation between S100A8/A9 and ESR was not statistically significant in our study even though it was close to the threshold of significance. We believe the reason for that is early sampling of our patients at the very beginning of the disease, while it is known that ESR is a somewhat slower marker of inflammation that requires some time to rise or fall. Similar correlations between serum S100A8/A9 levels and CRP, ESR, and ferritin were already established in other rheumatic diseases [26,27]. Complement is part of innate immunity. In the case of activation of the innate immune system, higher levels of C3 and C4 components of complement can be found in serum. Since innate response generates a high concentration of inflammatory cytokines, it is not unusual to have a correlation between serum S100A8/A9 levels and C3 levels, as in our study [28]. A correlation between serum S100A8/A9 and fibrinogen levels has already been shown in patients with systemic inflammation in coronary disease [29].

Elevated levels of S100A8/A9 that can be used as potential biomarkers in those diseases were also noticed in other autoimmune or autoinflammatory diseases such as juvenile dermatomyositis, inflammatory bowel disease, Behçet’s disease, polymyalgia rheumatica, cryopyrin-associated periodic syndromes, etc. [4,10,13], which makes us believe that the results of our study were not accidental.

Our study possesses various limitations, with the primary constraint being the relatively modest size of the patient cohort. Specifically, this pertains to the limited number of patients exhibiting a more severe clinical presentation and those with prolonged persistence of disease activity. Despite the study encompassing a comparatively small patient sample, a distinct correlation between the level of S100A8/A9 and disease activity is evident.

## 4. Materials and Methods

This prospective study was conducted in three pediatric tertiary centers in Croatia. The diagnosis of IgAV was established according to the criteria outlined by the European EULAR/PRINTO/PRES [3]. Data from medical records, including clinical information and laboratory parameters, were compiled.

Sex, age at disease onset, and manifestations in joints, kidneys, skin, and the gastrointestinal tract were considered in the clinical data. Laboratory findings encompassed erythrocyte sedimentation rate (ESR), C-reactive protein (CRP), leukocyte count, fibrinogen, serum albumin, gamma-globulins, ferritin, serum complement C3, and complement C4. For the purposes of this study, two additional laboratory parameters that were utilized were the levels of S100A8/A9 in both serum and fecal samples.

Serum, fecal, and urine samples were collected from 69 patients with IgAV at the onset of the disease (baseline), and then three and six months from the onset of the disease. Serum, fecal, and urine samples were also taken from 33 patients in the control group, which comprised children admitted to the hospital for reasons unrelated to autoimmunity, autoinflammation, or infectious diseases.

Samples were stored at −80 °C until analyzed. Analysis of serum and fecal S100A8/A9 was performed by enzyme-linked immunosorbent assay according to recommendations of the manufacturer, R&D Systems (R&D Systems, Inc., 614 McKinley Place NE, Minneapolis, MN, USA) [30].

To assess disease activity in patients with IgA vasculitis, a clinical questionnaire was employed, specifically the Pediatric Vasculitis Activity Score (PVAS). The PVAS comprises 64 clinical variables (symptoms or signs of the disease), categorized into nine organ systems. The evaluation involves determining which variables are new, have deteriorated in the past four weeks, or have persisted for no longer than three months. Each variable’s presence is assigned a specific number of points, which are then totaled [31].

Patients with active disease were defined as those patients with established diagnosis of IgAV that were having at least one sign of IgAV at the moment of evaluation (rash, arthralgia or arthritis, kidney disease, gastrointestinal involvement, etc.), and having PVAS 1 or higher at that point in time. The assessment was conducted at three specific time points: at the time of diagnosis, three months after the onset of the disease, and six months after the onset of the disease.

Patients with inactive disease (remission) were defined as those with no symptoms related to active vasculitis and had a PVAS score of 0 at a certain point in time: three months after the onset of the disease, and six months after the onset of the disease.

When we were assessing the correlation between skin affection and activity of disease, we divided the skin into four regions that were covered completely or partially with a rash: head and neck; trunk; arms; legs.

The primary goal was to determine the level of S100A8/A9 at different points in time, correlate it with disease activity, compare to patients who do not have inflammatory disease, and correlate it with other biomarkers of inflammation.

Statistical analysis was performed by Graphpad Prism 9.0 (GraphPad Software 225 Franklin Street. Fl. 26, Boston, MA, USA) and Past 4.16c software (free software available online: https://www.nhm.uio.no/english/research/resources/past/, (accessed on 20 March 2024)). Numerical data are described using medians and interquartile ranges, while categorical data are presented as absolute and relative frequencies. Variables were log-transformed in situations where distribution was skewed. Differences between groups were tested with an unpaired *t*-test with Welch’s correction and Mann–Whitney U test, ANOVA test, while tests for linear trends were used for trends between groups. *p*-values less than 0.05 were considered significant.

## 5. Conclusions

The role of serum S100A8/A9 in inflammation has been well known for some time now. It is a valid biomarker of activity of disease and, in some cases, even a prognostic factor in many rheumatic diseases, but also other inflammatory diseases and conditions. So far, it has not been widely researched in patients with IgAV, which comes as a bit of surprise since research of biomarkers in IgAV is a very active scientific topic nowadays.

In our study, we showed that serum levels of S100A8/A9 correlated well with disease activity and other biomarkers of inflammation in patients with IgAV. We believe that S100A8/A9 might be a potential biomarker of disease activity in this vasculitis. Further studies could prove this and also evaluate if this protein can be used as a predictor of disease flare up, as in some other autoimmune diseases.

## Figures and Tables

**Table 1:** Demographic characteristics of patients with IgA vasculitis, involvement of organs and number of patients with active disease in different observed periods of time.

**Characteristics of the Patients**	**N = 69**	**% of the Cohort**
**Demographics**		
Mean age (years)	6.4 (3.7–9.1)	
Female	33	47.8
Male	36	52.2
Ratio male/female	1.1:1	
**Clinics**		
Cutaneous manifestations ^1^	68	98.6
Joint involvement	56	81.2
Gastrointestinal involvement	26	37.7
Nephritis	18	26.1
**Active disease**		
Beginning of disease	69	100
After 3 months	9	13.0
After 6 months	5	7.2
**Treatment**		
Any treatment	61	88.4
Glucocorticoids	35	50.7
NSAID ^2^	42	60.9
Immunosuppressant	6	8.7
Antibiotics	9	13.0
Other	48	69.6
**Control Group**	**N = 33**	**% of the Cohort**
**Demographics**		
Mean age (years)	6.6 (3.8–9.2)	
Female	12	36.4
Male	21	63.6
Ratio male/female	1.75:1	

^1^ At the beginning of the disease. ^2^ NSAID—nonsteroidal anti-inflammatory drugs.

**Table 2:** Descriptive statistics of laboratory values tested in patients with IgA vasculitis and control group.

Parameter	Patients with IgA Vasculitis (Median, Interquartile Range)			Control Group (Median, Interquartile Range) (N = 33)	*p* *	*p* **	*p* ***	*p* ****	*p* *****	*p* ******
	Onset (N = 69)	Follow Up 3 Months (N = 63)	Follow Up 6 Months (N = 56)							
S100A8/A9 (ng/mL)	5348.00 (3171.00–8000.00)	2115.00 (1338.00–2725.00)	2013.00 (1196.40–4126.15)	1132.00 (797.50–2085.30)	**<0.0001**	**0.0086**	**0.0011**	**<0.0001**	**<0.0001**	0.3364
ESR (mm/h)	15.00 (9.00–23.00)	6.00 (4.00–9.00)	7.00 (5.00–10.75)	5.00 (4.00–9.25)	**<0.0001**	0.3540	0.0958	**<0.0001**	**<0.0001**	0.3866
CRP (mg/L)	5.50 (2.52–16.28)	0.70 (0.30–1.78)	0.90 (0.30–1.48)	0.00 (0.00–0.00)	**<0.0001**	0.1474	0.1010	**<0.0001**	**<0.0001**	0.5718
WBC (×10^9^/L)	10.10 (8.28–12.10)	7.30 (5.96–9.00)	6.95 (5.75–8.02)	5.50 (4.70–6.60)	**<0.0001**	**0.0028**	**0.0045**	**<0.0001**	**<0.0001**	**0.1173**
C3 (g/L)	1.35 (1.20–1.52)	1.16 (1.06–1.31)	1.19 (1.06–1.29)	1.05 (0.94–1.20)	**0.0062**	0.1288	0.1784	**0.0091**	**0.0058**	0.7504
C4 (g/L)	0.26 (0.20–0.33)	0.21 (0.16–0.25)	0.22 (0.16–0.26)	0.16 (0.13–0.24)	**0.0102**	0.1491	0.1532	**0.0072**	**0.0126**	0.9835
Fibrinogen (g/L)	3.40 (2.70–3.80)	2.60 (2.40–3.30)	2.70 (2.30–3.05)	2.55 (2.23–2.85)	**0.0181**	0.3822	0.7725	**0.0010**	**<0.0001**	0.2705
Albumin (g/L)	38.70 (35.63–41.92)	43.85 (40.65–45.48)	44.20 (42.00–46.70)	44.61 (40.65–47.10)	**0.0094**	0.4796	0.6462	**<0.0001**	**<0.0001**	**0.0479**
Gamma globulin (g/L)	9.60 (7.75–11.60)	9.03 (7.32–10.72	9.30 (7.50–10.90)	9.72 (8.24–12.02)	0.8685	0.6838	0.3377	0.5991	0.2053	0.4876
Ferritin (ng/mL)	64.00 (45.50–94.95)	22.80 (13.20–40.00)	28.00 (16.35–42.85)	31.50 (21.00–48.00)	**0.0448**	0.2951	0.0893	**<0.0001**	**<0.0001**	0.6486

Abbreviations: ESR—erythrocyte sedimentation rate; CRP—C-reactive protein; WBC—white blood cells. Legend: *p* *—comparison between values in patients at onset of disease and patients in control group; *p* **—comparison between values in patients three months after onset of disease and patients in control group; *p* ***—comparison between values in patients six months after onset of disease and patients in control group; *p* ****—comparison between values in patients at onset of disease and patients three months after onset of disease; *p* *****—comparison between values in patients at onset of disease and patients six months after onset of disease; *p* ******—comparison between values in patients three months after onset of disease and patients six months after onset of disease. Statistically significant values (*p* < 0.05) are bolded in table.

## Data Availability

The raw data supporting the conclusions of this article will be made available by the authors on request.
